# Understanding Dietary Protein Quality: Digestible Indispensable Amino Acid Scores and Beyond

**DOI:** 10.1016/j.tjnut.2025.07.005

**Published:** 2025-10-10

**Authors:** Joseph J Matthews, Emily J Arentson-Lantz, Paul J Moughan, Robert R Wolfe, Arny A Ferrando, David D Church

**Affiliations:** 1 Department of Geriatrics, Donald W. Reynolds Institute on Aging, Center for Translational Research in Aging and Longevity, University of Arkansas for Medical Sciences, Little Rock, AR, United States; 2 Department of Nutrition Sciences and Health Behavior, University of Texas Medical Branch, Galveston, TX, United States; 3 Center for Health Promotion, Performance and Rehabilitation Science, University of Texas Medical Branch, Galveston, TX, United States; 4 Riddet Institute, Massey University, Palmerston North, New Zealand

**Keywords:** protein, amino acids, digestibility, nutrition, diet, health

## Abstract

Dietary protein quality refers to the capacity of a food to meet the human metabolic needs for essential amino acids (EAAs) and nitrogen. This is critical in low- and middle-income countries, where severe protein malnutrition occurs, and relevant in higher-income countries, where increasing dietary EAA intake may improve health and function. There are several methods to assess protein quality, each with different objectives. Chemical scoring metrics, like the digestible indispensable amino acid score, describe the EAA composition and digestibility of a protein source. However, these methods do not capture the metabolic activity of food-derived amino acids. Overreliance on a single metric leads to generic dietary recommendations lacking individual context. This review draws on chemical score and stable isotope methods to provide a comprehensive assessment of dietary protein quality. We translate these findings into practical recommendations for improving protein quality in the context of whole diets. High-quality protein sources are characterized by high EAA density (%EAAs/kcals), digestibility, bioavailability, and the capacity to stimulate protein synthesis. Practically, protein quality improves when using processing and cooking methods that reduce antinutrients, denature proteins, and reduce food particle size and structure. Conversely, protein quality decreases when exposing foods to prolonged storage, heat sterilization, and high surface temperatures. Diet modeling studies show that EAA density and protein quality are higher in omnivorous and lacto-ovo-vegetarian diets, and diets high in whole food plant-derived proteins may require greater total protein and energy intakes to compensate for lower protein quality. For incomplete plant-derived proteins, consuming complementary proteins may be beneficial. Considerations for dietary protein quality in older adults include chewing efficiency, food particle size, and higher EAA density and leucine intakes to maximize muscle protein synthesis. Recognizing dietary protein quality as a multifaceted, modifiable metric is essential to improving dietary recommendations and public health outcomes.

## Introduction

The FAO defines dietary protein quality as the capacity of a food source to meet the metabolic needs for essential amino acids (EAAs) and nitrogen in humans [1]. This is determined by 3 main factors: human EAA requirements, the EAA composition of the food source, and the bioavailability of the ingested EAAs [2]. Protein quality is closely linked to protein quantity and the dietary reference intakes (DRIs), which reflect the minimum protein needed to maintain nitrogen balance. The estimated average requirement (EAR) and recommended dietary allowance (RDA) for adults are 0.66 g/kg/d and 0.83 g/kg/d, respectively [3]. EAR and RDA values are higher for children, pregnant, and lactating females [3], and expert groups recommend intakes of 1.0–1.5 g/kg/d for older adults (≥65 y) to minimize age-related declines in health and function [4,5]. The DRIs are primarily based on data from diets containing “high-quality” protein sources [6], defined as a protein digestibility-corrected amino acid score (PDCAAS) or digestible indispensable amino acid score (DIAAS) of 1.0 or ≥100%. Consuming the RDA for protein could lead to inadequacy if the protein source is of lower quality and does not provide sufficient bioavailable EAAs. In such cases, higher total protein intakes—or inclusion of higher quality protein sources—are necessary to achieve EAA requirements.

Dietary protein quality is a concern in many low- and middle-income countries (LMICs). In 2023, between 713 and 757 million people were estimated to be undernourished [7]. Food balance analysis showed that regions at the highest risk of protein inadequacy were in East and Southern Africa, West and Central Africa, Oceania, and South and South-East Asia [8]. The predicted prevalence of protein inadequacy increased further when intakes were adjusted for digestibility using either PDCAAS [8] or DIAAS [9]. Protein inadequacy was subsequently associated with a higher prevalence of stunting in children—a marker of protein malnutrition [8]. In these regions, diets rely primarily on protein from cereal grains such as sorghum, cassava, wheat, rice, or maize, which lack bioavailable EAAs, especially lysine, tryptophan [10,11], methionine, and leucine [12]. Consistent with this, stunted children in rural Malawi had EAA serum concentrations ∼10%–20% lower than nonstunted children, and lower concentrations of conditionally EAAs (arginine, glycine, and glutamine) and some non-EAAs (aspartate, glutamate, serine) [13]. These EAA deficiencies can become rate-limiting for protein synthesis, affecting growth, development, and infection risk [14].

In higher-income countries, where protein intakes typically exceed recommendations, the importance of protein quality based on its EAA composition has been questioned [15]. A recent analysis of NHANES data showed that ≥97% United States adults meet the RDA for protein [16]. However, when intakes were expressed as utilizable protein intake using DIAAS coefficients, the predicted prevalence of people with intakes of a limiting EAA below the RDA increased across all demographics—and up to 48% in adults ≥71 y [17]. Protein and EAA adequacy depend on the evidence behind DRIs, which are based on metabolic endpoints (nitrogen balance, amino acid balance, or isotope oxidation) rather than direct health outcomes. Although severe protein malnutrition is rare in higher-income countries, children raised on vegan diets with lower bioavailable EAA intakes may have decreased stature and bone mineral content compared with age-matched omnivores [18]. Evidence also supports protein quality and EAA intakes above the RDA for improving or maintaining strength, lean body mass [[19], [20], [21]], bone mineral density [22], and physical function [23]. These outcomes are important for groups with higher protein requirements and those with lower habitual bioavailable EAA intakes.

**FIGURE 1:**
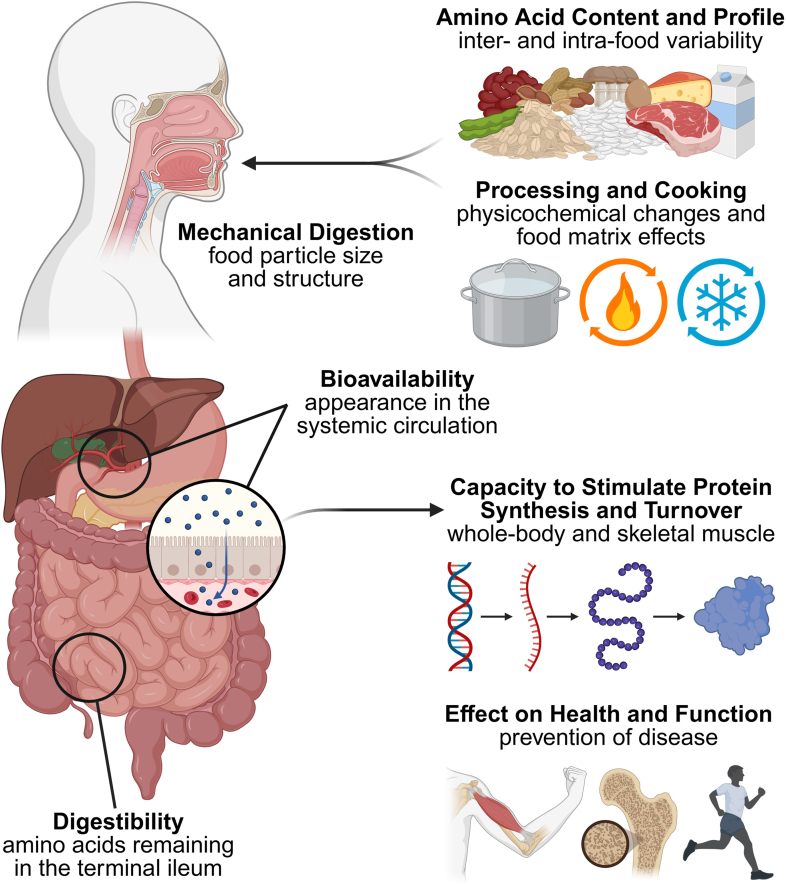
The determinants of dietary protein quality in humans. The food chemistry encompasses essential amino acid content, profile, and digestibility, which in turn can be modified by the food matrix, processing, cooking, and mechanical digestion. Bioavailable amino acids can contribute to protein synthesis and turnover. These processes may be up- or downregulated with certain dietary patterns and in different physiological states.

Despite the importance of protein quality, current understanding is limited by an overreliance on single PDCAAS or DIAAS values and labeling protein sources as “low” or “high” quality without additional context. This can result in generic, one-size-fits-all dietary recommendations. A complete picture requires contrasting the food chemistry underpinning protein quality with the metabolic activity of food-derived amino acids. From a food chemistry perspective, protein quality depends on the EAA composition, digestibility, food matrix interactions, and modifiable changes from cooking and processing. These factors influence amino acid absorption kinetics, bioavailability, and the capacity to stimulate protein synthesis (Figure 1). Therefore, this review draws on multiple chemical scoring and stable isotope methods to provide a comprehensive assessment of the determinants of dietary protein quality. Second, we translate these findings into practical recommendations for improving dietary protein quality in the context of whole diets.

## Protein Quality and Digestibility Metrics

The chemical scoring methods commonly used to assess protein quality focus on the digestible EAAs provided relative to a reference requirement pattern (Table 1) [1,3,24]. For regulatory purposes, the default EAA reference requirements used are for 6 m to 3 y olds [1]. However, the chemical score (AAS, PDCAAS, DIAAS) can be calculated for any reference pattern such as the EAA requirements for an adolescent, adult, or a custom reference pattern. In 2013, the FAO recommended replacing the PDCAAS with the DIAAS as the preferred measure of protein quality [1,25]. The DIAAS overcomes some limitations of the PDCAAS by *1*) accurately accounting for endogenous EAA turnover in the gastrointestinal tract; *2*) measuring the disappearance of EAAs at the terminal ileum [true ileal amino acid digestibility (TID)]; *3*) measuring digestibility values for individual EAAs, rather than a single value based on crude protein digestibility; *4*) measuring reactive digestible lysine rather than total digestible lysine; and *5*) permitting values ≥100% [(or ≥1.0 for digestible indispensable amino acid ratios (DIAARs)] to help compare higher scoring proteins. A PDCAAS or DIAAS score of 1.0 or ≥100% means that the food provides sufficient amounts of all EAAs, assuming all daily protein intake (based on the EAR: 0.66 g/kg/d) was supplied from this source [25]. Higher scores show the food provides a greater amount of digestible EAAs, indicating higher protein quality. TID is more accurate than the fecal protein digestibility method, which misrepresents amino acid absorption [26,27]. To further improve translation to humans, TID measurements should be made in humans, growing pigs, or growing rats, in that order [25,28]. For regulatory purposes, the DIAAS is based on a single limiting EAA; thus, low amounts of 1 EAA can truncate the score and hide its ability to provide large amounts of other EAAs. This can be overcome by interpreting the DIAARs for each individual EAA, although this approach is less common. The DIAAS does not describe absolute EAAs provided per portion, meaning that a food could have a high DIAAS score but may only contribute a small amount toward daily EAA intake, and vice versa. Lastly, the DRIs underpin all chemical scoring methods, which assume the current guidelines recommend sufficient EAAs to meet the body’s metabolic needs (for a critical review, see [29]).

**TABLE 1:** Commonly used protein quality and digestibility metrics.

Chemical scoring metrics	
AAS; amino acid score	The amino acid content in the food source relative to age-specific amino acid dietary reference intakes (e.g., for ≥18 y); not corrected for amino acid or protein digestibility AAS = (mg amino acid in 1g of the test protein)/(mg amino acid requirement in 1 g of the dietary reference protein intake) The amino acid with the lowest score is considered the first-limiting amino acid in the food source

DIAAR; Digestible indispensable amino acid ratio DIAAS; Digestible indispensable amino acid score	The relative amounts of dietary EAAs in the test protein, with each individual EAA corrected for true ileal digestibility (oro-ileal) and expressed relative to a profile of amino acid requirements DIAAR = [(mg digestible amino acid in 1 g of the test protein)/(mg amino acid requirement in 1g of the dietary reference intake)] DIAARs are calculated for all EAAs in the food source, and the lowest score (i.e., the first-limiting EAA in the food source) is the DIAAS; values may be reported as ratios or percentages (%) DIAAS (%) = 100 × [(mg the first-limiting digestible amino acid in 1 g of the test protein)/(mg amino acid requirement in 1 g of the dietary reference intake)] The dietary reference pattern typically used for regulatory purposes is the EAA requirements for 6 mo to 3 y olds

PDCAAS; Protein digestibility-corrected amino acid score	The relative amounts of dietary EAAs in the test protein, corrected for digestibility using a single value for true fecal crude protein digestibility (oro-fecal) and expressed relative to a profile of amino acid requirements PDCAAS (%) = 100 × true fecal crude protein digestibility × [(mg the first-limiting amino acid in 1g of the test protein)/(mg amino acid requirement in 1 g of the dietary reference intake)] Scores can be presented as coefficients (0–1) or percentages (0–100%); all scores are truncated to 1 (or 100%) so it is not possible to distinguish between protein sources that score ≥1 (or ≥100%)
Bioavailability and metabolic activity metrics	
IAAO; Indicator amino acid oxidation	Measures the metabolic activity (MA) of ingested EAAs by calculating the ratio of the response to the addition of EAA from the test protein to that of free (crystalline) EAAs using the slope oxidation method To ensure that the test EAA is first limiting for protein synthesis, the intake must be 1 or 2 SDs below the EAR in every individual MA (%) = 100 × (slope of ^13^C recovery for test protein/slope of ^13^C recovery for reference amino acid) Requires measurement of ^13^CO_2_ enrichment in breath samples

NPPU; Net postprandial protein utilization	Measures N retention and utilization via ingestion of intrinsically labeled (^15^N) dietary proteins corrected for true ileal N digestibility, and recovery of N in the body urea pool and urine NPPU (%) = 100 × (^15^N ingested – [^15^N in ileal digesta + ^15^N in urea + ^15^N in urine])/(dietary amino acid intake) Requires measurement of oro-ileal digestibility via oro-intestinal intubation

PPU; Postprandial protein utilization	Estimates protein utilization via a continuous intravenous infusion of a labeled amino acid (1-^13^C leucine) and measurement of leucine balance in response to 1 or 2 (low and high protein) meal feeding protocols Leucine balance is converted to N balance depending on leucine conversion factors of proteins and expressed with respect to meal N intake: PPU (%) = 100 × (N balance/N intake) Requires measurement of ^13^CO_2_ enrichment in breath samples and ^13^C enrichments in plasma samples

Dual isotope tracer method	Measures true ileal amino acid digestibility using 2 intrinsically labeled proteins, e.g., ^2^H-labeled test protein and ^13^C-labeled reference protein; can be used as a digestibility measurement to calculate DIAAS values TAAD = 100 × TCF × reference protein digestibility × [(plasma ^2^H-EAA/meal ^2^H-EAA)/(plasma ^13^C-EAA/meal ^13^C-EAA)] Requires measurement of ^13^C and ^2^H enrichments in the test meals and plasma samples

Method equations and interpretation from references [1,3,24].

Abbreviations: EAA, essential amino acids; EAR, estimated average requirement; IAA, indispensable amino acid; N, nitrogen; TCF, transamination correction factor.

Other methods use stable isotope tracers and intrinsically labeled dietary proteins to measure bioavailability and metabolic activity (Table 1). The FAO [25] recommends the indicator amino acid oxidation (IAAO) [30], net postprandial protein utilization (NPPU) [31], postprandial protein utilization [32], and the dual tracer approach for determining TID in humans [33] as “potentially” suitable methods. These provide different insights to the chemical scoring methods by measuring labeled amino acid appearance in the breath, plasma, urine, or feces (for a review, see [24]). Scores are expressed as percentages relative to the total amount of amino acid intake; that is, 100% equals complete bioavailability or utilization, indicating higher protein quality. Conversely, scores <100% show the proportion of ingested amino acid lost to digestion, absorption, or splanchnic utilization. These methods have some advantages; for example, because the IAAO slope method measures a relative change in net protein synthesis, it accounts for absorbed but nonutilizable amino acids [25,34]. The NPPU and dual tracer approaches can provide digestibility estimates for nearly all amino acids in a single trial, but the measurement of ileal nitrogen using naso-intestinal intubation, used in the NPPU, is an invasive technique unsuitable for routine application [25]. Other challenges include the use of intrinsically labeled proteins, an expensive and time-consuming process currently limited to a small selection of whole-food sources (e.g., beef, milk, cheese, chicken, eggs, wheat, soy, rapeseed, spirulina, chickpeas, yellow peas, faba beans, mung beans, and some of their corresponding protein isolates). Furthermore, some stable isotope labels (e.g., ^15^N) undergo dilution, uptake, and recycling in the gastrointestinal tract and splanchnic bed, leading to misrepresentation of exogenous rate of appearance and amino acid digestibility [35,36].

Chemical and stable isotope methods offer unique insights into different aspects of protein quality. DIAAS is the most widely used metric, and advances in measuring digestibility have improved the accuracy of these chemical scores, whereas stable isotope methods characterize the bioavailability and utilization of food-derived amino acids. To fully capture dietary protein quality, the following sections discuss EAA composition, digestibility, bioavailability, and metabolic activity in the context of these methods.

## Determinants of Dietary Protein Quality

### EAA composition and digestibility

**FIGURE 2:**
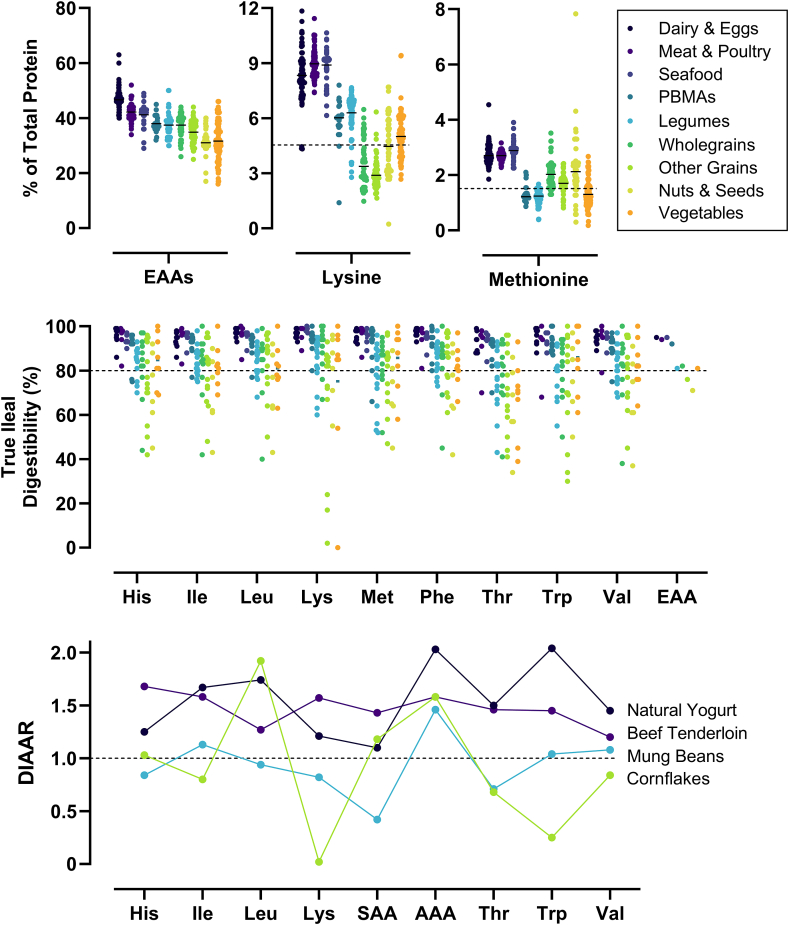
Essential amino acid (EAA) composition, true ileal amino acid digestibility (TID), and digestible indispensable amino acid ratio (DIAAR) profiles for various food groups. Top panel: EAA density of selected foods (*n* = 884) from the USDA FoodData Central database; foods in cooked or ready-to-eat form. Dashed line represents the dietary requirements for lysine and methionine as a proportion of total protein [3]. The “other grains” category includes refined and processed grains. Middle panel: TID of selected foods (*n* = 79 foods from *n =* 19 countries). Measurements from sampling the terminal ileum in growing pigs, humans, or using the dual stable isotope method in humans. Dashed line represents 80% digestibility, whereby a portion 25% larger is required to match fully digestible EAAs. Bottom panel: DIAARs for representative foods across 4 food groups (dairy, meat, legumes, grains). Values calculated from the reference EAA requirements for 6 mo to 3 y olds. Dashed line represents 1.0, whereby consumption of the protein source in line with the estimated average requirement (0.66 g/kg/d) would be sufficient to supply all dietary EAAs. Data from refs [[41], [42], [43], [44], [45], [46]]. AAA, aromatic amino acids (methionine and cysteine); PBMAs, plant-based meat alternatives; SAA, sulfuric amino acids (phenylalanine and tyrosine).

EAA content varies across food groups, from as low as 0.07 g EAAs per 100 g for applesauce to ∼42 g EAAs per 100 g for whey protein isolate [37,38]. Variations also exist within food groups, due to genotype, cultivar, dry mass, processing, and cooking. For example, the total EAA content per 100 g of varies by up to 50% depending on the location and trimming of the cut, with even larger relative differences among cheese products and types of nuts and seeds [37]. Large within-product variation was shown in 20 breakfast cereals, where the coefficient of variation for individual amino acids ranged from 6% to 48% for different batches of the same cereal [39]. Furthermore, the protein content of the dry matter in 59 pea types was shown to range from 14% to 31% [40]. Despite this, there are consistent trends across food groups when expressing EAAs relative to total protein (Figure 2) [3,[41], [42], [43], [44], [45], [46]].

Animal-derived proteins generally contain sufficient amounts of each EAA (relative to daily EAA requirements), making them “complete” protein sources. In contrast, plant-derived proteins often lack sufficient amounts of 1 or more EAAs, making them “incomplete” protein sources. Cereals, grains, and seeds tend to be proportionally low in lysine, whereas legumes and vegetables are proportionally low in the sulfuric amino acid, methionine (Figure 2). Most plant-derived proteins also contain limited amounts of valine and isoleucine [47]. Some vegetables have a high proportion of EAAs relative to total protein (spinach: ∼50% EAAs/total protein) but provide few EAAs on a per portion basis (0.74 g per 100 g spinach). Thus, they have limited practical utility as a protein source. When plant-derived foods cannot provide EAAs in sufficient quantities, consideration should be given to balancing EAA profiles across foods, termed “protein complementation” [48]. Several IAAO studies have shown that feeding complementary proteins (rice with chickpeas or lentils, and lentils with millet or sorghum) in a mixed meal increases the metabolic availability of lysine and methionine [[49], [50], [51], [52]]. Plant-based meat alternatives (PBMAs)—like tofu, seitan, mycoprotein, and soy products—have favorable EAA profiles (Figure 2). On a per-portion basis, natto, tempeh, mycoprotein, and soy-based meat alternative (SBMA) products provide ∼6–7 g EAAs per 100 g, comparable to equivalent portions of whole eggs [37]. On the basis of EAA content alone, these foods may be regarded as high-quality protein sources, although this does not necessarily translate to bioavailability and metabolic activity, discussed later in the article.

EAA digestibility also varies across food groups. Animal-derived proteins and PBMAs generally have high EAA digestibility (≥90% TID), meaning that the EAA composition is close to what the body will absorb. Legumes, wholegrains, and vegetables have good EAA digestibility (≥80%), although consideration should be given to processed grains and specific foods with low digestibility, such as corn-based products (tortillas; 54%). Nuts and seeds have the lowest mean EAA digestibility (∼71%) as a food group, but this hides high TID of individual foods (cashews: 90%) and the variability of EAA digestibility within foods (lysine in sunflower seeds: 94%, and threonine in chia seeds: 34%) [41]. The variable digestibility for plant-derived foods is due to more complex food matrices (e.g., proteins located within intact fibrous cell walls) and the presence of antinutrients such as protease inhibitors, polyphenols, oxalates, saponins, tannins, phytates, and fiber [53]. These compounds form indigestible complexes with proteins and inhibit digestive enzyme action, thereby reducing or delaying EAA digestion and absorption.

The food matrix encompasses the physical structure and organization, nonprotein ingredients (e.g., lipids, carbohydrates, fiber, micronutrients, water, and other bioactive compounds), and interactions between nutrients and non-nutrient components. Separating plant-derived proteins from their original food matrix, creating protein concentrates and isolates, can improve EAA density and digestibility. For example, the DIAAS for green peas ranges from 61% to 100% depending on whether they are consumed as a whole food [41]; protein concentrate, with some native carbohydrates and antinutrients [38]; or protein isolate, largely separated from the original food matrix [54]. Furthermore, soy and potato protein isolates both have DIAAS ≥100% when calculated using the reference pattern for children and adults (≥3 y) [55]. This gives them comparable protein quality to animal-derived whey protein isolates (DIAAS: 94%–100%) [38,56], although still well below casein isolates (DIAAS: 145%) [54]. Thus, plant-derived proteins with favorable EAA composition but unfavorable EAA digestibility can be modified to improve their protein quality, making them valuable sources for vegan and vegetarian diets.

### Processing and cooking

Most foods undergo processing, be it mild (e.g., removal of inedible materials), moderate (e.g., soaking, fermentation, dehulling, milling, curing, or pasteurization), or more extensive (e.g., enzyme treatments, separation, and reincorporation into a new food product) [57]. Foods may then be heated via boiling, baking, frying, grilling, or steaming. Insights from TID, DIAAS, PDCAAS, and the IAAO methods show how these steps alter protein quality by increasing or decreasing EAA digestibility and metabolic activity (Supplemental Table 1).

**FIGURE 3:**
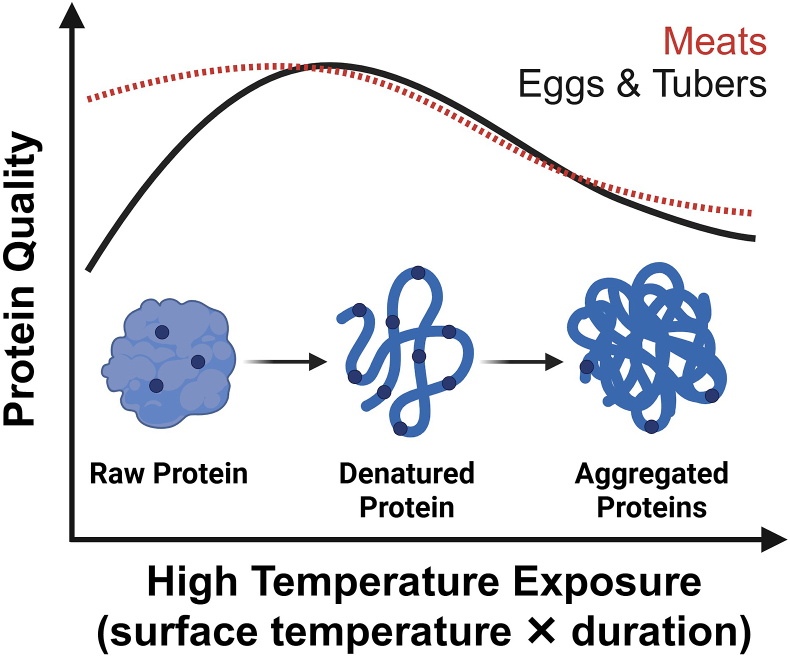
Processing and cooking effects on protein quality. The solid line represents the typical relationship between cooking and protein quality for some foods (i.e., eggs and tubers). The dashed red line represents the effects for some meat products (i.e., beef) where digestibility is similar when uncooked and with moderate cooking.

Common household processing methods in LMICs, such as soaking, dehulling, and fermentation, can increase the protein quality of plant-derived sources. Dehulling removes indigestible fibers and tannin-rich outer layers of the seed coat, whereas soaking reduces protease inhibitors, leading to improved protein quality in mung beans, soybeans, and other legumes [58,59]. Fermentation reduces phytates and tannins while increasing endogenous microbial protease activity, which helps to hydrolyze proteins into smaller peptides and free amino acids [60]. For foods like eggs and potatoes, moderate cooking increases amino acid availability by up to 2-fold, and by up to one-third for mung beans cooked as a dal [59,[61], [62], [63]]. This occurs due to a breakdown of antinutritive factors that interfere with protein digestibility and amino acid absorption in raw foods. For example, cooking egg whites causes structural changes in proteins (e.g., ovalbumin and ovotransferrin) and inactivates protease inhibitors (e.g., ovomucoid and ovoglycoprotein) present within raw eggs [64]. Moderate cooking temperatures improve the digestibility of some foods by causing protein denaturation, leading to conformational changes that expose cleavage sites to digestive enzymes and increase pepsin action via hydrolysis (Figure 3) [65]. A series of studies showed that extrusion, cooking, and baking reduced heat-sensitive antinutritive factors and improved the DIAAS for beans, chickpeas, lentils, and milled pulses [[66], [67], [68], [69]]. Cooking improved the DIAAS for Amadeus soy protein, but not for Etna soy and wheat [70], indicating some crop-specific effects.

Processing and cooking can also decrease protein quality by reducing digestibility and metabolic activity. EAAs are generally heat-stable, and direct losses from soluble protein fractions leaching into boiling liquid tend to be minimal [71,72]. Cooking can induce substantial changes, evidenced by the DIAAS for beef steaks ranging from 80% to 121% [42,73] (Supplemental Table 1). This can be explained by higher surface temperatures with grilling (225^o^C) than with boiling (80^o^C), pan-frying (186^o^C), and roasting (160^o^C). Similarly, well-cooked ground beef (internal temperature >72^o^C) had lower digestibility and DIAAS than raw and medium-cooked ground beef [73,74]. Rodent studies support this, showing that longer cooking times and high-temperature methods like deep-frying reduce potato protein digestibility [61]. Another study showed no difference in overall digestibility with veal cooked to 3 temperatures (60^o^C, 75^o^C, and 95^o^C), although higher temperatures caused delayed plasma EAA appearance and a lower area-under-the-curve from 0 to 3 h post meal [75]. Overall, there is a relationship between prolonged exposure to high surface temperatures during cooking and lower (or slower) EAA digestibility. For proteins with a tertiary structure, this occurs due to irreversible conformational changes that include protein oxidation and carbonylation, leading to cross-link formation and protein aggregation [65,74,75]. Figure 3 shows how these steps conceal digestive enzyme cleavage sites and reduce digestive enzyme action.

Processing and cooking may affect digestibility only slightly but result in a substantial proportion of the amino acid being absorbed in a modified, nonutilizable form. Certain EAAs are more susceptible to these changes due to reactive, free amino groups on their side chain. The Maillard reaction, which causes lysine glycation, commonly occurs in dairy, cereals, grains, and legumes [34,76,77]. Lysine glycation reduces postprandial plasma lysine availability [78]; thus, only “reactive” lysine is metabolically available. With heat sterilization and storage, reactive lysine in bovine and soy milk can be high (∼94%–99% of total lysine) but drops substantially in some plant-derived milk alternatives: oat (49%), rice (40%), almond (73%), and coconut (69%) [79]. Reduced lysine availability especially affects grains and cereals, where lysine is often the first-limiting EAA. Figure 2 shows an example of cornflakes, where a low amount of reactive lysine results in a DIAAS of 2%. Tryptophan, threonine, and methionine are also susceptible to oxidation, phosphorylation, and other treatment effects that reduce their metabolic availability [80]. Despite this, assays to determine utilizable proportions of each EAA are not routinely used, and postprocessing modifications may not be accounted for in calculations of TID. Collectively, these data show the modifiable nature of protein quality across foods. A single DIAAS value cannot be representative of a whole food group, such as DIAAS for beef or for potatoes. The large variation from routine processing and cooking means there are useful practical strategies that maintain or increase the protein quality of many animal- and plant-derived foods.

### EAA bioavailability

**FIGURE 4:**
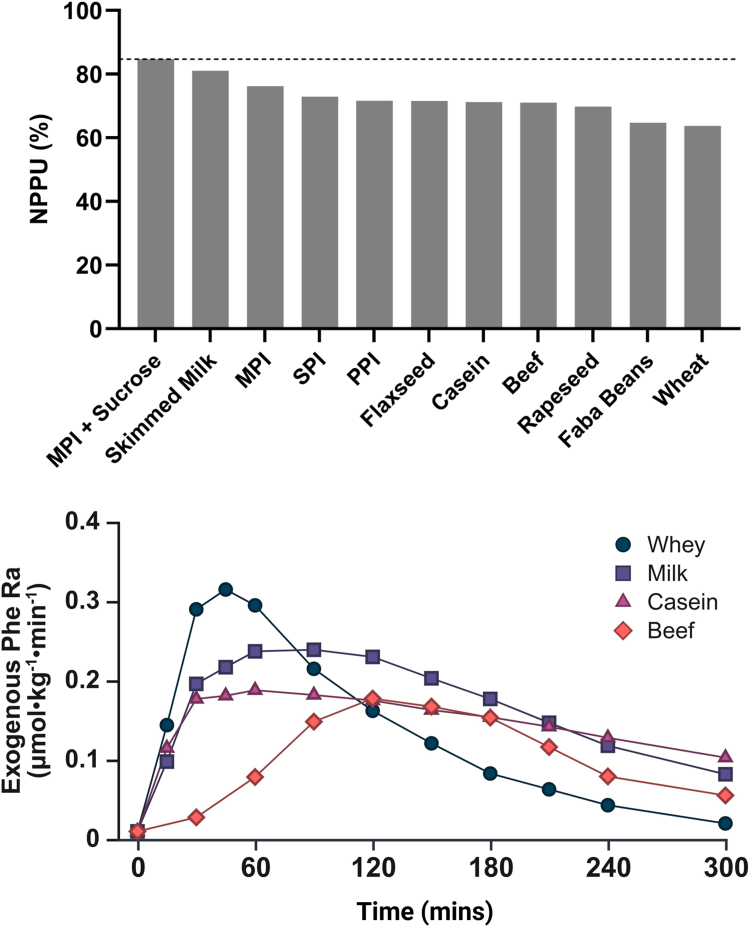
Dietary protein sources and essential amino acids bioavailability. Top panel: Net postprandial protein utilization (NPPU) across foods. Proteins were typically consumed as part of a mixed meal; flaxseed, rapeseed, and wheat proteins were incorporated into biscuits. Data from refs: [45,54,74,[81], [82], [83], [84], [85], [86], [87], [88]]. Bottom panel: rate of appearance (*R*a) of ^15^N-labeled phenylalanine from 3 bovine protein isolates and bovine whole food sources: whey protein concentrate, micellar casein, milk protein concentrate (∼20% whey and ∼80% casein proteins), and cooked mince beef. Data redrawn from refs: [89,90]. MPI, milk protein isolate; PPI, pea protein isolate; SPI, soy protein isolate.

Bioavailability encompasses protein and EAA digestibility, absorption in an utilizable form, and its freedom from interference in metabolism that limits its utilization in the body [1]. DIAAS and PDCAAS were not designed to describe the metabolic effects of bioavailable EAAs. Instead, stable isotope methods using labeled ^15^N proteins can measure the rate of appearance of food-derived amino acids in the systemic circulation and their overall retention in the body. Studies using the NPPU method show that 61%–85% of ingested nitrogen is bioavailable after accounting for ileal and deamination losses (Figure 4) [45,54,74,[81], [82], [83], [84], [85], [86], [87], [88], [89], [90]]. Data are only available for a limited number of foods, but when protein isolates are directly compared, soy-derived amino acids undergo greater deamination to urea than milk-derived amino acids [81]. Generally, around 25%–50% of EAAs, but ∼60%–90% of the EAA threonine, are taken up by the splanchnic tissues, as evidenced from postprandial measurements of the portal drained viscera in growing piglets [[91], [92], [93]]. Consequently, dietary proteins with a higher proportion of EAAs relative to non-EAAs generally have greater peripheral bioavailability [94,95].

Figure 4 shows the effect of the food matrix on postprandial kinetics and bioavailability. Milk-derived proteins, partly separated from their original food matrix, differ in their rate of appearance in plasma due to protein structure and solubility. For example, casein proteins naturally form large, stable micellar structures that coagulate in the stomach, resulting in slower gastric emptying and delayed amino acid absorption. Whey proteins are soluble globular proteins that do not form micelles or gels, and their compact structure allows for rapid digestion and absorption [96]. In both cases, amino acid digestion and absorption remain faster than with comparable whole-food sources (e.g., beef), where the intact food matrix leads to slower digestion and EAA rate of appearance in the plasma [89]. For labeled casein, whey, and milk protein isolates, the exogenous phenylalanine rate of appearance over a 5-h period was 45%, 57%, and 65% of total dietary intake (∼25 g protein) [89]. Dietary nitrogen not accounted for at this point was either not digested and absorbed, retained and taken up by the splanchnic tissues, or oxidized. This shows the variability in EAA plasma appearance and overall nitrogen retention; the proportion of EAAs reaching the peripheral circulation can influence the capacity of the food to stimulate protein synthesis.

### Capacity to stimulate protein synthesis and turnover

Dietary protein provides the EAA precursors to stimulate protein turnover. Achieving net protein accretion (or positive protein balance) requires synthesis to exceed breakdown. Muscle and whole-body protein synthesis (MPS and WBPS) increase in a dose-dependent manner after protein intake [97,98], and responses are greater in young, healthy, anabolically sensitive individuals, and after exercise [99]. Commonly used protein quality metrics (Table 1) do not assess this fundamental role of dietary protein. Here, we discuss the variables needed for a protein source to stimulate protein turnover and how this applies to protein quality.

Work from our group showed that, across a variety of protein sources, the proportion of EAAs relative to total protein had the strongest relationship with the change in MPS (*r* = 0.585) and WBPS (*r* = 0.409). This also correlated with the peak plasma EAA concentration, which was the best independent predictor of the delta and postprandial MPS response [100]. Of the EAAs, leucine is the most potent stimulator of the mammalian target of rapamycin (mTOR) anabolic signaling pathway [101]. Small doses of leucine-enriched EAAs (1.5–3.6 g EAAs, containing 0.6–1.3 g leucine) stimulate MPS equivalent to larger protein doses (15–40 g whey protein), despite markedly different postprandial kinetics [[102], [103], [104]]. This is beneficial in older adults, where higher leucine doses in an EAA or protein drink can overcome anabolic resistance to maximally stimulate MPS [105,106]. However, a sustained MPS response and net muscle protein accretion requires sufficient availability of all EAAs.

When matched for total EAAs or leucine content, liquid protein drinks from a range of sources cause similar increases in MPS: mycoprotein (mycelium-derived protein), milk protein, pea protein, corn protein, potato protein, pea and mycoprotein blend, corn and milk blend, and algae-derived proteins (chlorella and spirulina) [[107], [108], [109], [110], [111], [112]]. Studies comparing whole foods and protein isolate food matrices with their matched free-EAAs also show similar MPS responses [113,114]. Importantly, these dietary proteins all had high digestibility and caused substantial postprandial plasma aminoacidemia (≥50% above baseline). Whole-body protein balance can differ in response to liquid protein drinks. Whey protein intake led to higher nonoxidative leucine disposal (indicative of protein synthesis), whereas casein protein intake led to comparably lower protein synthesis, but greater suppression of protein breakdown and overall net positive protein balance [115]. Protein source absorption kinetics may therefore dictate whether the meal-derived amino acids are incorporated into muscle or other body tissues.

**FIGURE 5:**
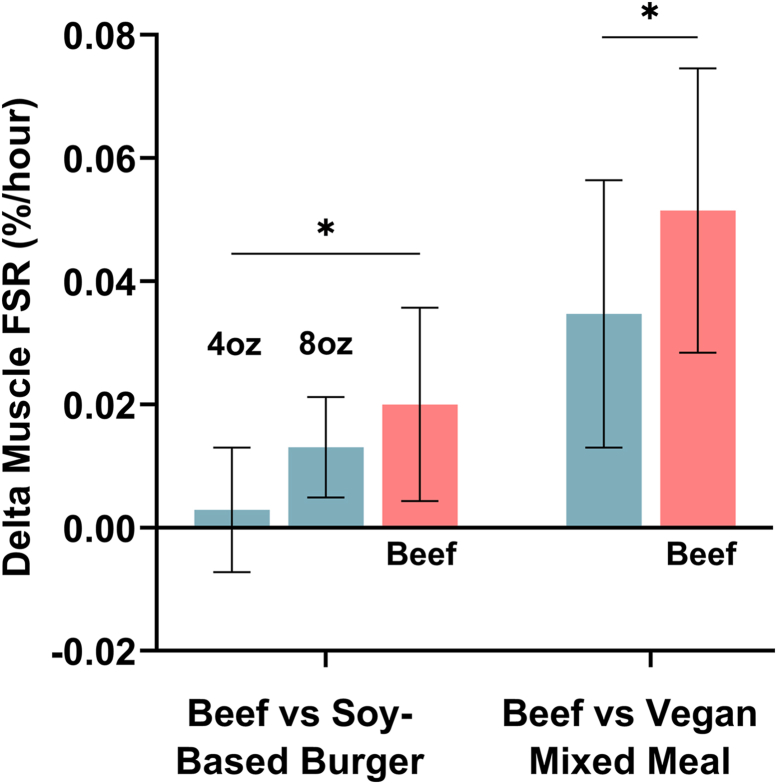
Muscle fractional synthetic rate (FSR) change from baseline to postprandial (0–6 h). Left: response to a 4oz beef burger and 4oz and 8oz soy-based meat alternative burger. Right: response to an isocaloric and isonitrogenous beef-based omnivorous meal and a vegan meal (protein sources: soybeans, chickpeas, quinoa). Data are presented as mean ± SD. ∗*P* < 0.05. Data extracted from refs: [116,117].

Most dietary protein intake comes from whole foods consumed within a mixed meal. MPS and WBPS responses differ from liquid protein drinks due to slower gastric emptying and absorption kinetics, and a larger insulin response (when co-ingested with carbohydrates). We showed that a 4oz beef burger led to a greater change in MPS than a 4oz SBMA burger, and similar MPS to an 8oz SBMA burger [116] (Figure 5). However, whole-body net protein balance was similar between the 4oz beef and 4oz SBMA groups [116], which may indicate greater splanchnic tissue uptake of slowly digested or soy-derived proteins, in line with our previous discussion. A similar study reported a 47% greater MPS response to a beef-based omnivorous meal compared with an isocaloric and isonitrogenous vegan meal (soybeans, chickpeas, quinoa) [117] (Figure 5). In both cases, the beef-derived proteins led to greater postprandial plasma EAA availability, providing a strong anabolic signal for MPS. The plant-derived meals were ∼10%–20% lower in leucine and EAAs, and higher in fiber, which can reduce EAA digestibility and absorption. It is worth noting that the beef burger and SBMA have comparable DIAAS (ground beef: 91%–99%, SBMA: 91%) [42,118], highlighting the inability of chemical scoring metrics to predict the muscle anabolic response. These data show that the total protein content of a food or meal does not explain the metabolic activity of the derived amino acids. Protein quality, therefore, should include the capacity of a food or drink to stimulate muscle and whole-body protein turnover on a per-gram or per-portion basis, dependent on the EAA density, leucine content, and postprandial kinetics.

## Protein Quality in the Context of Aging

Older adults appear to absorb and utilize whole food dietary proteins less effectively than younger adults. In whole food studies, protein bioavailability of minced beef was ∼5%–10% lower in elderly (≥70 y) than in younger adults, partly due to greater nitrogen loss to urea [74,119]. Denture wearers had slower postprandial leucine appearance and lower WBPS after eating beef steak compared with older adults with natural dentition [120]. Consistent with this, older adults absorbed amino acids faster and had greater whole-body net protein balance after eating minced beef compared with steak [90]. This can be partly explained by reduced chewing efficiency—determined by bite force, number of teeth, number of chewing cycles before swallowing, and the food particle size distribution—as well as reduced gastric acid secretion and slower gastric emptying [121]. Together, these changes impair protein digestion and delay amino acid absorption. This shows the importance of the food matrix; older adults with reduced chewing efficiency may consider foods with smaller particle sizes (e.g., minced beef compared with steak) or semisolids (e.g., yogurt or quark) to be higher protein quality sources.

In studies using liquid protein drinks, where chewing efficiency is not a factor, age-related differences in postprandial kinetics still occur. Following whey protein concentrate and free-form EAA intake, the first-pass splanchnic extraction of oral leucine and phenylalanine was shown to be higher in older adults (Phe: 47% ± 3%, Leu: 50% ± 11%) compared with young, healthy controls (Phe: 29% ± 5%, Leu: 23% ± 2%) [94,122]. In a large analysis of labeled protein isolate (whey, casein, milk) feeding studies, peak protein digestion and phenylalanine absorption rates were similar between young and older men [89]. However, rates declined more rapidly in older men, resulting in lower postprandial availability of dietary protein-derived phenylalanine in the circulation over 5-h: 45% ± 10% compared with 51% ± 14%. This translates to ∼1.3 g less of the ingested (∼22 g) protein appearing in the circulation of older adults [89]. Early studies showed that differences in splanchnic extraction did not affect arterial phenylalanine concentrations, delivery to the leg muscles, or overall MPS [122], suggesting the higher extraction may indicate a higher splanchnic turnover rate rather than reduced peripheral availability. It is worth noting that there is high variability in the extraction of dietary leucine, ranging from 26% to 88% in elderly men [94] and 18% to 63% in elderly women [123]. This may not be a function of aging per se, but we speculate that this could also reflect general metabolic health, whereby insulin resistance, inflammation, and other comorbidities necessitate greater splanchnic uptake of dietary amino acids to support protein synthesis and turnover of gut and liver tissues. Thus, the digestibility and metabolic activity of dietary protein and EAAs are likely to be more variable in older adults.

## Protein Quality in Whole Diets: Practical Considerations

**FIGURE 6:**
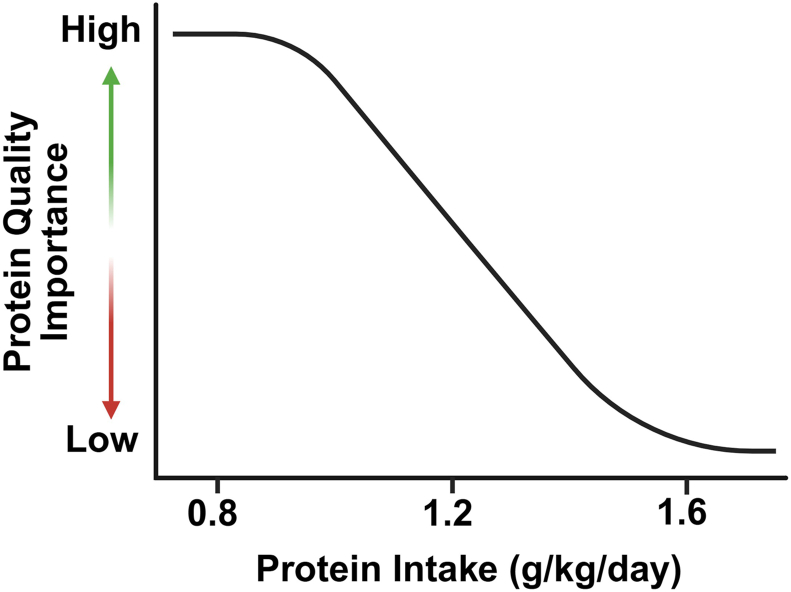
Relationship between protein quality and quantity (figure adapted from ref: [124]). Protein intake at the level of the recommended dietary allowance (0.8 g/kg/day) necessitates higher protein quality to achieve the benefits associated with essential amino acids (EAA) intake. As total daily protein intake increases, EAA intakes can be achieved with the inclusion of lower-quality protein sources.

In whole diets, individual EAAs intakes must be met on a per-meal or per-day basis. With lower daily protein intakes, higher protein quality is needed to meet EAA requirements and the associated health benefits (Figure 6) [124]. As daily protein intake increases beyond the RDA, EAA intakes can be more easily met from a range of dietary protein sources. Consistent with this, and to account for utilizable protein intake, the Dutch national dietary guidelines recommend a 30% increase in daily protein intake for people consuming vegan diets [125].

**FIGURE 7:**
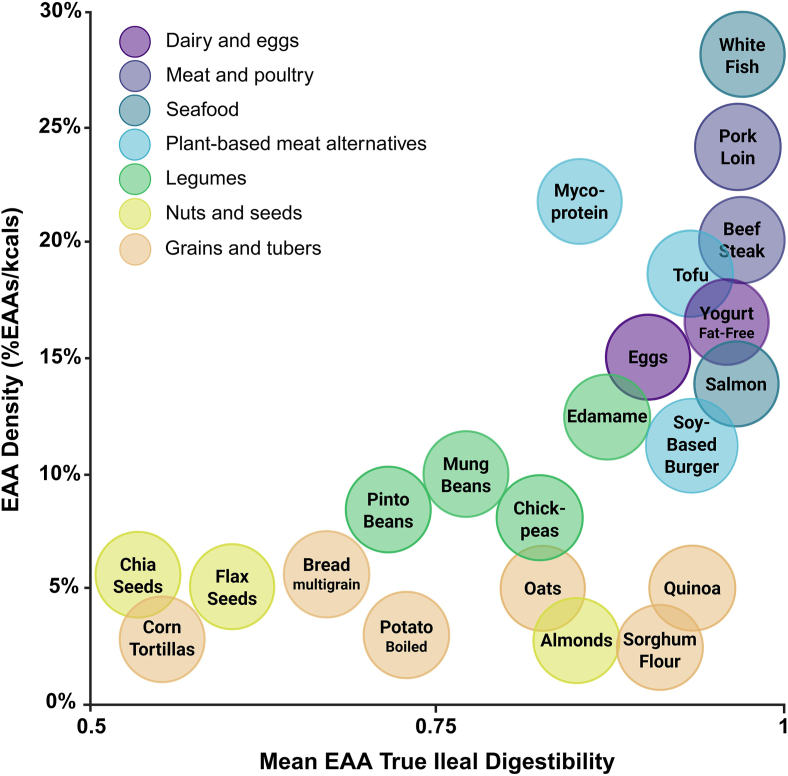
Essential amino acids (EAA) density (%EAAs/kcals) and mean EAA true ileal digestibility of common dietary protein sources. A high EAA density means that the food provides a higher proportion of EAAs per kcal. The protein sources displayed represent a range of foods groups to illustrate the EAA density concept, it is not an exhaustive list. White fish is representative of bass, catfish, or tilapia. Mycoprotein represents mycelium-derived Quorn pieces. Data derived from refs: [37,41,42,46,59].

We have discussed EAA density (%EAAs/kcals) as an important feature of high-quality proteins (Figure 7). Diet modeling studies show that EAA density is higher when including animal-derived proteins, that is, in omnivorous or lacto-ovo-vegetarian diets [126,127], whereas diets high in plant-derived proteins typically have lower EAA density, and require greater total protein and energy intakes [127]. This is important for groups with lower energy needs, such as older sedentary women who may only require ∼1600 kcal/d. In this case, a whole food vegan diet requires ∼300 kcal/d extra to meet age-related protein intakes of 1.3 g/kg/d [126]. It is worth noting that these were diets designed by nutrition professionals with skills and knowledge to optimize food combinations and EAA intakes. Another strategy is to include PBMAs or plant-derived protein isolates that have digestibility and EAA density similar to some animal-derived sources (Figure 7). One concern is that PBMAs may not have equivalent capacity to stimulate MPS, and higher total protein intakes may still be needed to maximize anabolism [116,128]. A recent meta-analysis showed that animal-derived proteins may have a small beneficial effect over non-soy proteins for increasing strength and lean body mass [129]. However, studies in highly active young and healthy older adults show that when daily protein intake is sufficient (1.2–1.8 g/kg/d), vegan diets containing plant-derived protein isolates and PBMAs have similar rates of MPS [130], and gains in lean body mass and strength, compared with omnivorous diets [131,132]. This does not occur when the additional protein comes from collagen, a low-quality protein source (∼18% of protein from EAAs), compared with high-quality pea (∼39%) and whey (∼50%) protein isolates [133]. If it is feasible in the target population, adding high EAA density protein sources to diets based on low-quality proteins is an effective way to increase dietary protein quality.

Protein quality methods assess individual foods (Table 1). Two novel tools, the EAA-9 and the meal protein quality score (MPQS), aim to quantify EAA intake across a meal or day in relation to individual needs [134,135]. The EAA-9 functions as a cumulative measure of the amino acid score (AAS), whereas the MPQS focuses on a personalized per-meal basis (e.g., containing 0.3 g/kg/meal for an older adult) to account for the theoretical timeframe for consuming complementary proteins to maximize their metabolic activity. When applied under these conditions to a sample of Dutch participants (*n* = 5121 meals), the MPQS showed that no vegan meals (*n* = 357) achieved a score of 100 (100 = all EAAs were adequately provided in the meal), and the limiting EAAs were (lysine, 79% of meals), methionine (20%), and leucine (1%) [135]. Similarly, in a German cohort, intake of all EAAs was significantly lower (median difference –22.9% to –51.9%) in vegans compared with omnivores [136]. Although it is sometimes possible for a well-structured vegan diet to meet protein and EAA requirements, this shows that there may be a knowledge or behavior gap in the general population. The EAA-9 and MPQS rely on the PDCAAS and do not account for how cooking and processing affect protein quality. Despite this, these tools represent a step toward assessing protein quality of dietary patterns, and their use has the potential to improve our understanding of protein quality intake and distribution.

Protein quality across multiple meals and days is linked to the complementary proteins concept. It is thought that if complementary proteins are not consumed within a certain timeframe, then the insufficient EAA becomes rate-limiting for protein synthesis, causing deamination or oxidation of other excess EAAs. Debates over the timeframe for consuming complementary EAAs include within the same meal, within 3 h, within a single day, and within sequential days [136]. We discussed that feeding complementary proteins (rice with chickpeas or lentils, and lentils with millet or sorghum) in a mixed meal increases the metabolic availability of lysine and methionine [[49], [50], [51], [52]]. This supports the notion that a deficiency of a single EAA within a meal becomes rate-limiting for protein synthesis. However, a recent study reported similar 24-h MPS responses to complete (28.5 g EAAs), complementary (20 g EAAs), and incomplete (16.5 g EAAs) protein diets [137]. Skeletal muscle and whole-body protein breakdown were not measured, so it is unclear whether net synthesis was similar or if the incomplete protein diet caused the breakdown of endogenous proteins to provide EAA precursors for MPS. It is possible that the body compensates for insufficient EAAs in the short term from plasma or muscle free amino acid pools or by releasing endogenous EAAs from rapidly turning-over gut tissues [138,139]. However, over the long term, this would lead to greater protein breakdown, loss of whole-body proteostasis, and potentially lean body mass. Understanding the time course of complementary EAA intake will enable better practical guidance to people following vegan diets and in LMICs, where complementing protein sources (e.g., beans with rice) may not always be culturally appropriate.

Protein quality and the metabolic activity of food-derived amino acids may depend on the background or habitual diet. The body appears to adapt to low protein intake by upregulating amino acid transporters, altering protein turnover, or improving nitrogen retention. The postprandial bioavailability of milk, whey, wheat, and soy protein was reduced by 5%, 5%, 10%, and 13%, respectively, after short-term habituation to a high protein diet (1.5–2 g/kg/d) compared with a control protein diet (0.7–1 g/kg/d) [82,83,140]. Current metrics encompass food matrix effects, but we know less about mixed-meal effects that occur through the co-ingestion of protein sources with other nutrients. For example, the TID of whole boiled egg protein decreased by 17% when co-ingested with black tea, a beverage high in polyphenols [43]. In contrast, co-ingestion of a pea protein isolate supplement with microbial proteases improved postprandial plasma aminoacidemia, indicating better digestibility and absorption [141]. It is unclear whether this translates into practically meaningful changes. Several studies in growing pigs show EAA digestibility is the same when food is given as a single feed as part of various mixed diets, and the TID of individual EAAs appears to be additive over a wide range of feedstuffs [142,143]. Given these uncertainties, dietary protein quality in the context of mixed meals and whole diets is a valuable avenue for future research.

## Protein Quality beyond Current Metrics

We have discussed dietary protein quality in relation to EAA composition, bioavailability, and metabolic activity. This definition does not consider other potentially beneficial or detrimental health effects of protein-rich foods. Many animal-derived protein foods are rich sources of bioavailable micronutrients and nonprotein nitrogen compounds. For example, creatine, carnitine, carnosine, and β-alanine have been shown to improve metabolic, musculoskeletal, and cognitive health [[144], [145], [146]]. Plant-derived protein foods improve health via large amounts of micronutrients, fiber, polyphenols, and phytonutrients. Isolating nutrients from their native food matrix improves EAA bioavailability. However, this comes at the expense of beneficial effects of the whole-food matrix such as the protein–mineral–lipid matrix, which can enhance EAA metabolic activity [147]. High-fiber foods, despite reducing digestibility, provide precursors for short-chain fatty acid production may influence skeletal muscle anabolism [148]. Some PBMAs have high EAA density and protein quality, making them valuable for vegan diets, although they may undergo ultraprocessing methods with potentially detrimental health effects [149]. Substituting PBMAs for products with similar nutrition fact label content (e.g., beef burger compared with soy-derived burger) does not mean the protein sources are nutritionally interchangeable. Untargeted metabolomic analyses showed a 90% difference in metabolite abundances in cooked grass-fed beef compared with a PBMA [150]. Even within a food source, a 58% difference in metabolites and lipids was detected between beef from pasture- compared with grain-finished cattle [151]. This highlights the diversity in protein food sources, with the health effects of these additional compounds largely unknown. Protein quality is just 1 nutrition metric, and a higher quality protein source is not inherently better for overall health. Instead, food choices must be considered as part of a whole diet, alongside other beneficial and potentially harmful components of protein-rich foods.

## Conclusions

Understanding dietary protein quality requires insights from chemical scoring metrics and stable isotope methods. Collectively, these describe the food chemistry and the metabolic activity of food-derived amino acids. This enables interpretation beyond single metrics, like the PDCAAS or DIAAS. We have evaluated how dietary protein quality is variable, influenced by EAA composition, bioavailability, and processing and cooking methods. Table 2 summarizes our main discussion and provides practical recommendations to improve dietary protein quality in whole diets. These recommendations depend on the population and context. For example, older adults experiencing anabolic resistance may benefit from protein sources rich in leucine and foods with smaller particle sizes. People in LMICs at risk of protein malnutrition require changes to food systems to provide greater quantities of plant-derived proteins, protein sources with complementary or complete EAA profiles, alongside the use of household processing methods to improve digestibility. People consuming vegan diets may benefit from high EAA density sources, like PBMAs or plant-derived protein isolates, and consuming complementary protein sources. Despite recent advances, our understanding of protein quality is based on a limited selection of foods and protein isolates. The DIAAS represents an improvement on previous chemical scoring methods, and its utility will grow as more individual EAA digestibility data become available. Variation in protein and EAA content and digestibility remains a challenge for protein quality assessment, especially when using methods like food frequency questionnaires (i.e., those used to collect national dietary intake data). Emerging tools like the EAA-9 and MPQS represent promising approaches to assessing protein quality at the whole-diet level, but they require further refinement to incorporate cooking effects, food matrix interactions, and real-world dietary patterns. Recognizing dietary protein quality as a multifaceted, modifiable metric is essential to improving dietary recommendations and public health outcomes.

**TABLE 2:** Practical recommendations to improve dietary protein quality in whole diets.

• Include foods with higher EAA density (% of total kcals)
• Consume foods with complementary EAA profiles to increase their metabolic activity
• Use cooking methods that minimize prolonged high surface temperatures
• Avoid prolonged heat sterilization and prolonged storage of liquid plant-derived proteins
• Remove fibrous, indigestible components of food (e.g., dehulled mung beans)
• Reduce antinutrient content of foods via soaking, sprouting, and fermentation
• Include foods high in leucine, particularly in older adults
• Effective chewing (mastication) of food before swallowing
• Prioritize foods with smaller particle sizes in older adults with reduced dentition
• Reduce food particle size/structure before cooking it within a mixed meal (e.g., dal)

Abbreviation: EAA, essential amino acids.

## Author contributions

The authors’ responsibilities were as follows - JJM & DDC: jointly conceived and designed the work; JJM wrote the article; and all authors: read, critically reviewed, and approved the final manuscript.

## Funding

The author(s) declare that no financial support was received for the research, authorship, and/or publication of this article.

## Conflict of interest

EJA-L reports a relationship with U.S. Dairy Export Council that includes: funding grants, speaking and lecture fees, and travel reimbursement; National Cattlemen’s Beef Association that includes: funding grants, speaking and lecture fees, and travel reimbursement; and National Pork Board that includes: speaking and lecture fees and travel reimbursement. PJM reports a relationship with U.S. Dairy Council that includes: funding grants; National Cattlemen’s Beef Association that includes: funding grants; United States Department of Agriculture (USDA) that includes: speaking and lecture fees; and Global Dairy Platform that includes: speaking and lecture fees. AAF reports a relationship with United States Army Medical Research and Development Command (USAMRAA) that includes: funding grants; Department of the Army, US Army Research Institute of Environmental Medicine that includes: funding grants; Air Force Office of Scientific Research that includes: funding grants; National Pork Board that includes: funding grants; National Dairy Council that includes: funding grants; and has patent issued to University of Arkansas for Medical Sciences. RRW reports a relationship with National Cattlemen’s Beef Association that includes: funding grants; National Cattlemen’s Beef Association that includes: speaking and lecture fees; has patent issued to The Amino Company; and is a founder and part owner of The Amino Company. DDC reports a relationship with National Institutes of Health that includes: funding grants; National Pork Board that includes: funding grants; National Cattlemen’s Beef Association that includes: funding grants; Soy Connection funded by U.S. Soy that includes: consulting or advisory; Shifted Supplements that includes: board membership; Protein PACT that includes: travel reimbursement; National Dairy Council that includes: funding grants. If there are other authors, they declare that they have no known competing financial interests or personal relationships that could have appeared to influence the work reported in this paper.
