# Supplementary material for: Understanding Dietary Protein Quality: Digestible Indispensable Amino Acid Scores and Beyond
Source: J Nutr. 2025 Oct 10;155(10):3152–67. doi: 10.1016/j.tjnut.2025.07.005 (PMC12799415; doi:10.1016/j.tjnut.2025.07.005)
Supplement: multimedia component 1 [file mmc1.docx]

| Supplemental Table 1. The effects of processing and cooking on protein quality. | | |
| --- | --- | --- |
| *Food (ref)* | ***Form and processing methods*** | ***Effect on protein quality*** |
| Pork  (1) | Pork loin convection oven-cooked to three internal temperatures (63^o^C, 68^o^C, 72^o^C)  Cured bacon: smoked vs smoked-cooked (microwave)  Ham cooked: non-cured, conventionally cured^b^, or alternatively cured^c^ | Medium-rare cooked (63^o^C) had a higher DIAAS (129%) compared with other temperatures: 68^o^C and 72^o^C (both: 109%)  Smoked-cooked had a higher DIAAS (126%) compared with smoked (109%)  Alt-cured ham had a higher DIAAS (123%) compared with other curing methods: non-cured (115%) and con-cured (117%)  Similar TID between all processing methods |
| Beef  (2)  (3) | Ribeye steaks smoke house-cooked to three internal temperatures (56^o^C, 64^o^C, 72^o^C)  Ground beef: raw vs well-cooked (72^o^C)  Topside steaks: raw, boiled, grilled, pan-fried, or roasted (internal temperatures: 71^o^C) | Medium-cooked (64^o^C) had a higher DIAAS (121%) compared with other temperatures: 56^o^C (104%) and 72^o^C (99%)  Raw ground beef had a higher DIAAS (111%) compared with well-cooked beef (92%)  Decreased EEA content and DIAAS in grilled steaks (80%) compared with other cooking methods: raw (97%), boiled (99%), pan-fried (98%), or roasted (91%)  Similar TID between all processing methods |
| Eggs  (4)  (5)  (6) | Egg whites (raw vs microwave-cooked)  Whole eggs (raw vs hard-boiled)  Fried whole eggs  Boiled whole eggs  Scrambled whole eggs  Prepared with firm yolks (minimum internal temperatures: 71^o^C) | Raw eggs TID: 51%  Cooked eggs TID: 91%  Hard-boiled eggs:  54% ↑ postprandial aminoacidemia (indicating higher EAA bioavailability)  Similar DIAAS across cooking methods:  Fried whole eggs: 114%  Boiled whole eggs: 110%  Scrambled whole eggs: 118% |
| Milk  (7) | Protein isolates: milk concentrate vs whey concentrate vs whey isolate | Similar digestibility with all methods (96-97%)  DIAAS highest for milk concentrate (120%; limited by SAA) compared with whey isolate and concentrate (100% and 107%; limited by histidine) |
| Corn (maize)  (8,9) | Yellow maize (ground) vs cornflakes (processing: tempering, cooking, rolling, and toasting) | Yellow maize SID: 80%  Cornflakes TID: 69%  Yellow maize DIAAS: 48% (lysine)  Cornflakes DIAAS (2%; reactive lysine) |
| Mung beans  (10)  (11) | Hulled vs dehulled mung beans (both soaked and pressure-cooked)  Whole mung beans vs mung bean dal (dehulled and split beans ground with a food processor) | Whole mung beans TID: 63%  Dehulled mung beans TID: 71%  (24% ↓ in fibre; 64% ↓ in polyphenols)  Whole mung beans TID: 64%^a^  Mung bean dal TID: 89%^a^ |
| Nuts  (12) | Pistachio nuts: raw vs roasted | Similar EAA composition and TID with small differences in DIAAS: raw (86%) and roasted (83%) |
| Potatoes  (13) | Russet potatoes: raw, microwaved, baked, boiled, and fried | True fecal protein digestibility lower for raw (41%) compared with all other methods: microwaved (83%), baked (85%), boiled (83%), and fried (80%)^a^  PDCAAS was lowest for raw (27%) and highest for boiled (56%) compared with other methods: microwave (49%), baked (50%), and fried (36%)^a^ |
| Rice  (14) | Long-grain white rice: boiled or boiled then oven-cooked (browned) | IAAO metabolic activity of lysine: cooked white rice (97%) and oven-browned cooked rice (70%) |
| Soybeans  (7,15) | Processed soybeans: tofu, soya milk (UHT), and soy protein isolate (SPI) | Similar TID with all methods (93-94%)  DIAAS highest for soya milk (99%; limited by lysine) and similar for tofu and SPI (83% and 84%; limited by SAA) |
| DIAAS, digestible indispensable amino acid score using 6-month to 3-year-old reference values; EAA, essential amino acids; PDCAAS, protein digestibility corrected amino acid score; TID, true ileal digestibility; SAA, sulphuric amino acids (methionine and cysteine); SID, standardized ileal digestibility; UHT, ultra-high-temperature. ^a^Measured in growing rats; ^b^Cured using sodium-nitrate and sodium-chloride (pink curing salt); ^c^Cured using nitrate-free sodium-chloride (celery salt). | | |

**References**

1. Bailey HM, Mathai JK, Berg EP, Stein HH. Pork products have digestible indispensable amino acid scores (DIAAS) that are greater than 100 when determined in pigs, but processing does not always increase DIAAS. J Nutr Elsevier BV; 2020;150:475–82.

2. Bailey HM, Mathai JK, Berg EP, Stein HH. Most meat products have digestible indispensable amino acid scores that are greater than 100, but processing may increase or reduce protein quality. Br J Nutr Cambridge University Press (CUP); 2020;124:14–22.

3. Hodgkinson SM, Montoya CA, Scholten PT, Rutherfurd SM, Moughan PJ. Cooking conditions affect the true ileal digestible amino acid content and digestible indispensable amino acid score (DIAAS) of bovine meat as determined in pigs. J Nutr Elsevier BV; 2018;148:1564–9.

4. Evenepoel P, Geypens B, Luypaerts A, Hiele M, Ghoos Y, Rutgeerts P. Digestibility of cooked and raw egg protein in humans as assessed by stable isotope techniques. J Nutr Elsevier BV; 1998;128:1716–22.

5. Fuchs CJ, Hermans WJH, Smeets JSJ, Senden JM, van Kranenburg J, Gorissen SHM, Burd NA, Verdijk LB, van Loon LJC. Raw eggs to support post-exercise recovery in healthy young men: Did rocky get it right or wrong? J Nutr Elsevier BV; 2022;152:2376–86.

6. Fanelli NS, Martins JCFR, Stein HH. The digestible indispensable amino acid score (DIAAS) in eggs and egg-containing breakfast meals is greater than in toast breads or hash browns served without eggs. J Nutr Sci 2024;13:e68.

7. Mathai JK, Liu Y, Stein HH. Values for digestible indispensable amino acid scores (DIAAS) for some dairy and plant proteins may better describe protein quality than values calculated using the concept for protein digestibility-corrected amino acid scores (PDCAAS). Br J Nutr Cambridge University Press (CUP); 2017;117:490–9.

8. Cervantes-Pahm SK, Liu Y, Stein HH. Digestible indispensable amino acid score and digestible amino acids in eight cereal grains. Br J Nutr Cambridge University Press (CUP); 2014;111:1663–72.

9. Hodgkinson SM, Stroebinger N, Stein HH, Fanelli N, de Vries S, van der Wielen N, Hendriks WH, Moughan PJ. True ileal amino acid digestibility of human foods classified according to food type as determined in the growing pig. Journal of Nutrition.

10. Kashyap S, Varkey A, Shivakumar N, Devi S, Reddy B H R, Thomas T, Preston T, Sreeman S, Kurpad AV. True ileal digestibility of legumes determined by dual-isotope tracer method in Indian adults. Am J Clin Nutr Elsevier BV; 2019;110:873–82.

11. Rutherfurd SM, Bains K, Moughan PJ. Available lysine and digestible amino acid contents of proteinaceous foods of India. Br J Nutr Cambridge University Press (CUP); 2012;108 Suppl 2:S59-68.

12. Bailey HM, Stein HH. Raw and roasted pistachio nuts (Pistacia vera L.) are “good” sources of protein based on their digestible indispensable amino acid score as determined in pigs. J Sci Food Agric Wiley; 2020;100:3878–85.

13. Bailey T, Franczyk AJ, Goldberg EM, House JD. Impact of cooking on the protein quality of Russet potatoes. Food Sci Nutr 2023;11:8131–42.

14. Prolla IRD, Rafii M, Courtney-Martin G, Elango R, da Silva LP, Ball RO, Pencharz PB. Lysine from cooked white rice consumed by healthy young men is highly metabolically available when assessed using the indicator amino acid oxidation technique. J Nutr Elsevier BV; 2013;143:302–6.

15. Reynaud Y, Buffière C, Cohade B, Vauris M, Liebermann K, Hafnaoui N, Lopez M, Souchon I, Dupont D, Rémond D. True ileal amino acid digestibility and digestible indispensable amino acid scores (DIAASs) of plant-based protein foods. Food Chem 2021;338:128020.
